# Sesame Oil (*Sesamum Indicum L.*) as a New Challenge for Reinforcement of Conventional Glass Ionomer Cement, Could It Be?

**DOI:** 10.1155/2021/5516517

**Published:** 2021-03-22

**Authors:** Neven S. Aref

**Affiliations:** ^1^Department of Dental Biomaterials, Faculty of Dentistry, Mansoura University, Mansoura, Egypt; ^2^Department of Basic Oral and Medical Sciences, College of Dentistry, Qassim University, Buraydah, Saudi Arabia

## Abstract

**Purpose:**

Despite the advantages of glass ionomer cement (GIC) including chemical bonding to the tooth structure and fluoride release, its low-grade mechanical properties make it a topic for research. Accordingly, this study was conducted to assess the ability of sesame oil as a natural bioactive additive to reinforce conventional glass ionomer cement.

**Materials and Methods:**

Sesame oil was blended into the liquid component of the cement in ratios of 3 and 5 (v/v%). One control and two experimental groups were enrolled in the study; I: unmodified GIC (control), II: 3 (v/v%) sesame oil-modified GICs, and III: 5(v/v%) sesame oil-modified GICs. Compressive strength, shear bond strength, diametral tensile strength, surface microhardness, surface roughness, and color stability were the parameters assessed. A representative specimen of each group was analyzed for its chemical structure by Fourier transformation infrared spectroscopy. One-way ANOVA followed by Tukey test was used to analyze the collected data of all evaluated parameters except the color stability results, which were analyzed by Student *t*-test at *p* < 0.05.

**Results:**

Three and 5 (v/v%) sesame oil-modified GICs exhibited significant increase in their compressive strength, shear bond strength, diametral strength, and surface microhardness. Concurrently, there was a significant decrease in surface roughness (*p* < 0.05) in both formulations of the modified cement. Both 3 and 5 (v/v%) sesame oil-modified GICs showed a clinically acceptable color change.

**Conclusions:**

Sesame oil seems to be a promising natural bioactive product for reinforcement of conventional GIC with a clinically agreeable esthetic.

## 1. Introduction

Two main resinous restorative materials, resin composites and glass ionomers, are being used as alternatives to mercury-containing restoration due to its deleterious effects [1]. These materials represent revolutionary white restorative materials. To date, glass ionomer cements (GICs) have been known as materials of choice due to their unique physical and biological criteria [2].

These properties include an excellent coefficient of linear thermal expansion/contraction and modulus of elasticity, as well as being the only restorative material able to bond chemically to the tooth structure, anticariogenic properties, biocompatibility, and fluoride release and uptake, which contributes to its preventive character [2, 3]. Additionally, contrary to composites, they possess negligible shrinkage upon setting. Yet, their poor mechanical properties in terms of low fracture strength, toughness, and wear resistance make them of a lesser use in the stress bearing areas and a subject of improvement [4]. So, as to overcome these limitations, a variety of modifications have been applied to the cement powder and liquid, such as bioactive apatite, zirconia, silica, zinc, fibers, strontium oxide, and nanocrystals. Consequently, glass ionomer cement may now be indicated for posterior and anterior restorations of deciduous and permanent teeth [5–7].

An example of the trials of modification is the incorporation of bioactive component as nanohydroxyapatite into GIC. Such modification considerably enhanced the compressive, tensile, and flexural strength of the set cement. The bioactive crystals had the ability to improve the chemical stability and decrease the water solubility resulting in better performance of the cement compared to conventional one. The enhanced mechanical properties were due to the ability of apatite crystals to strongly bond to the polyacrylic acid of the cement and to the calcium ions of the tooth structure [8, 9].

Recently, the antimicrobial activity of many natural oils has been proven through different in-vitro studies and among these oils is sesame oil. Sesame is a valuable oilseed crop that contains a mixture of bioactive compounds including lignans, tocopherol homologues, and phytosterols. Sesame oil (*Sesamum indicum L*.) contains three lignans: sesamine, sesamolin, and sesaminol, that exhibit excellent antioxidant properties and accordingly the oil is commonly known as Queen of Oil seeds. It has vitamin E, and certain amounts of unsaturated fatty acids able to reduce the free radical injury of the oral tissues. Sesame seed is rich in protein, vitamin B_1_, and dietary fiber. Additionally, it is an excellent source of phosphorous, iron, magnesium calcium, manganese, copper, and zinc. In addition to its bioactive composition, sesame oil proved to possess an antibacterial property through its viscosity plus an emulsification process. Accordingly, it is able to diminish the bacterial adhesion to the tooth structure with an enhancement of oral hygiene [10–12].

Although previous studies [13, 14] investigated the influence of sesame oil on reducing plaque accumulation and dentin hypersensitivity depending on its antibacterial and antioxidant activities, however, no researches inspected its bioactive composition impact on the properties of dental restorative materials. So, in the light of enhanced mechanical properties of the previously modified GIC with bioactive materials as nanohydroxyapatite and the bioactive composition of sesame oil, this study intended to assess the ability of sesame oil to reinforce conventional GIC. The null hypothesis was that sesame oil would not be able to either reinforce conventional GIC or influence its esthetic.

## 2. Materials and Methods

A conventional glass ionomer restorative material (GC Corporation, Tokyo, Japan) and Sesame oil (FLORA, INC., LYNDEN, WA98264, USA) were used in the study. The oil was incorporated into the liquid of the cement in ratios of 3 and 5 (v/v%). The mixtures were kept on the magnetic stirrer for 24 hours for obtaining a homogenous mix. The utilization of these different liquid formulations produced three different groups: control and two experimental groups, as follows:

  Group I: unmodified GIC
  Group II: GIC modified by 3 (v/v%) sesame oil
  Group III: GIC modified by 5 (v/v%) sesame oil

Hence, a total no. of 90 specimens were prepared for assessment of compressive strength, shear bond strength, diametral tensile strength, surface microhardness, surface roughness, and color stability [15], a specimen for each test, and 5 specimens for each group. A representative sample of each group and a sample of sesame oil were explored for their chemical structure by Fourier transformation infrared spectroscopy (FTIR).

Powder and liquid of each formulation were mixed and specimens were prepared, cured, finished, and polished, in keeping with the manufacturer instructions and the specifications assigned for each test. All specimens were kept in deionized water at 37°C for 24 hours before testing.

### 2.1. Fourier Transformation Infrared Spectroscopy

Chemical structure of the specimens was analyzed using a spectrometer (Nicolet iS10, America). Solid specimens were ground into fine powder, mixed with KBr, and pressed into a homogenous disc to be investigated and data were recorded in the range of 500–4000 cm^−1^.

### 2.2. Compressive Strength

Cylindrical specimens of 4 mm in diameter and 6 mm in length were prepared in a split Teflon mold. Compressive strength was assessed by a Universal Testing Machine (Model 3345, Instron Corporation, Canton, MA, USA). The compressive load was applied to the specimen at a crosshead speed of 1 mm/min until fracture. Compressive strength (CS)(MPa) was calculated according to the following formula:

(1) $$\begin{array}{c} \text{CS}=\frac{4P}{\pi D^{2}}, \end{array}$$

where *P* is the maximum applied load at fracture (*N*) and *D* is the diameter of the specimen (mm).

### 2.3. Shear Bond Strength

Fifteen sound third molar teeth (extracted due to impaction) were used for preparing the specimens. After scaling and cleaning of the musing with a sharp scaler and chloramine-T, molars had their occlusal surface cut to below the dentinoenamel junction. The exposed flat dentine surface was smoothened using silicon carbide paper. The roots of the molar were mounted in acrylic blocks. The exposed dentin surface was conditioned by polyacrylic acid for 20 sec. A split Teflon mold 4 mm × 4 mm was used for bonding GIC to the dentin surface. A shear load was directed to the bonding interface using the Universal Testing Machine (Model 3345, Instron Corporation, Canton, MA, USA) at a crosshead speed of 1 mm/min. The shear bond strength (MPa) was determined as follows:

(2) $$\begin{array}{c} \tau =\frac{P}{\pi r^{2}}, \end{array}$$

where *τ* is the shear bond strength, *P* is the load at failure (*N*), and *r* is the radius of the specimen (mm).

### 2.4. Diametral Tensile Strength (DTS)

Cylinders of 6 mm in length and 4 mm in diameter were prepared in a split Teflon mold. Specimens were tested by the same Universal Testing Machine at cross head speed of 0.5 mm/min. Specimens were positioned horizontally and were compressed diametrically introducing tensile stress in the material perpendicular to the vertical plane passing through the center of the specimen. Diametral tensile strength (MPa) was calculated as follows:

(3) $$\begin{array}{c} \text{DTS}=\frac{2P}{\pi \;DT}, \end{array}$$

where *P* is the load applied (*N*), *D* is the diameter of the cylinder (mm), and *T* is the thickness of the cylinder (mm).

### 2.5. Surface Microhardness

A split Teflon mold was used for preparing disc-shaped specimens of 6 mm × 3 mm. By means of a Vickers microhardness tester (HMV Microhardness Tester, Shimadzu, Japan), a load of 50 g was employed by the diamond indenter for a dwell time of 10 sec. For each specimen, five measurements were registered and the average value was recorded. The Vickers hardness number (VHN) was calculated and expressed in kg/mm^2^.

### 2.6. Surface Roughness (Ra)

Disc-shaped specimens of dimensions 8 mm × 2 mm were employed for surface roughness estimation by a surface profilometer (Mitutoyo Surf Test SJ 210 Analyzer; Mitutoyo Corp, Japan). Five measurements at different locations for each specimen were obtained and the average was calculated. The stylus speed was 0.5 mm/s and the tracing length was 8 mm.

### 2.7. Color Stability

Discs from each group were prepared in a split Teflon mold of 8 mm × 2 mm. A spectrophotometer (Vita Zahnfabrik H. Rauter GmbH and Co. KG, Bad Sackingen, Germany) was used to assess the color coordinates (*L*^*∗*^, *a*^*∗*^, *b*^*∗*^) for each specimen. Determined CIE *L*^*∗*^, *a*^*∗*^, and *b*^*∗*^ for each specimen was compared to those of the control specimen and the color difference (Δ*E*) was estimated as follows:

(4) $$\begin{array}{c} \Delta E={\left[{\left(\Delta L^{*}\right)}^{2}+{\left(\Delta a^{*}\right)}^{2}+{\left(\Delta b^{*}\right)}^{2}\right]}^{1/2}, \end{array}$$

where *L*^*∗*^ is the color value (lightness) and *a*^*∗*^ and *b*^*∗*^ represent chromaticity. Color difference (Δ*E*) ≤ 3.3 is clinically acceptable.

Collected data were statistically analyzed using analysis of variance (ANOVA) and Tukey test, for compressive strength, diametral tensile strength, surface microhardness, and surface roughness at a significance level of 0.05. Student *t*-test used to analyze the color stability results.

## 3. Results

**Figure 1:**
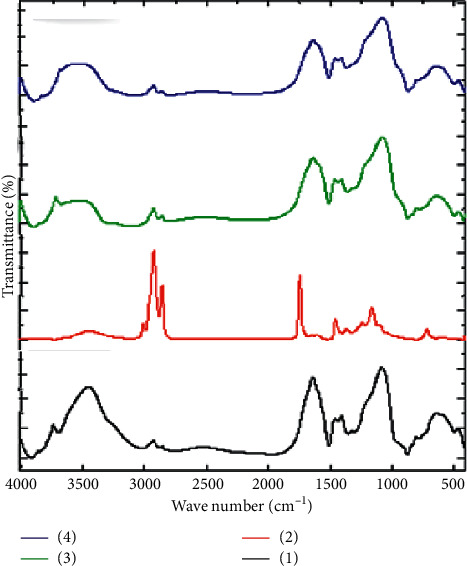
FTIR spectra of GIC, sesame oil, 3 (v/v%) sesame oil-modified GIC and 5 (v/v%) sesame oil-modified GIC.

Figure 1 presents FTIR spectra of GIC, sesame oil, 3 (v/v%) sesame oil-modified GICs, and 5 (v/v%) sesame oil-modified GICs. On modification of glass ionomer cement with sesame oil in both ratios, 3 and 5 (v/v%), the intensity of the absorbance peak at 3738 cm^−1^ decreased with minor shift to 3718 cm^−1^ and excessive shift to 3687 cm^−1^ in 3 and 5 (v/v%) sesame oil-modified GICs, respectively. Additionally, the same change was detected for the peak at 3542 cm^−1^ indicating less sharp peak with an obvious shift to 3512 cm^−1^ in both ratios of modification. The absorbance peaks at 2926, 1640, 1410, and 1085 cm^−1^ were nearly the same with no variations distinguished. There was a minor shift at the peak 627 to 638 cm^−1^ in 3 and 5 (v/v%) sesame oil-modified GICs.

Results of compressive strength, diametral tensile strength, surface microhardness, and surface roughness are shown in Table 1. For compressive strength (MPa), it seems that sesame oil GIC modified by 5 (v/v%) had the highest mean (130.34 ± 4.15) while the unmodified cement had the lowest (116.42 ± 3.02). ANOVA indicated a significant difference among groups (*p* ≤ 0.05). Tukey test revealed that both 3 and 5 (v/v%) sesame oil-modified groups were significantly different from the unmodified one. On the other hand, no significant difference was detected between 3 and 5 (v/v%) sesame oil-modified groups (*p* ≤ 0.05).

Shear bond strength (MPa) of 3 (v/v%) sesame oil-modified GIC had the highest mean value (7.03 ± 0.38) while the control group had the lowest mean (5.08 ± 0.68). ANOVA revealed a significant difference among groups (*p* ≤ 0.05). Tukey test indicated that both experimental groups, 3 and 5 (v/v%) sesame oil-modified GIC, were significantly different from the unmodified cement, yet, no significant difference was recognized between the two experimental groups (*p* ≤ 0.05).

Regarding diametral tensile strength (MPa), modification of GIC with sesame oil significantly increased the DTS with the highest mean belonging to 5 (v/v%) sesame oil-modified GICs (5.9 ± 0.53). ANOVA showed a significant difference among groups (*p* ≤ 0.05). Post-hoc test disclosed that both 3 and 5 (v/v%) sesame oil-modified cement were significantly different from the unmodified GIC. Conversely, no significance was noticed between both experimental groups, 3 and 5 (v/v%) sesame oil-modified GIC (*p* ≤ 0.05).

With reference to surface microhardness (kg/mm^2^) results, unmodified cement had the lowest value (52.84 ± 1.22) while 3 (v/v%) sesame oil-modified GICs had the highest value (60.08 ± 0.65). ANOVA analysis proved a significant difference among groups (*p* ≤ 0.05). Tukey test verified that both 3 and 5 (v/v%) sesame oil-modified groups were significantly different from the unmodified GIC. Quite the reverse, no significance was noticed between both test groups, 3 and 5 (v/v%) sesame oil-modified GIC (*p* ≤ 0.05).

On the subject of surface roughness (*μ*m), the lowest mean was for 3 (v/v%) sesame oil-modified cement (0.34 ± 0.07) while the unmodified cement got the highest mean (0.8 ± 0.09). A significant difference was observed among groups by ANOVA (*p* ≤ 0.05). Tukey test demonstrated that all groups were significantly different from each other (*p* ≤ 0.05).

**Figure 2:**
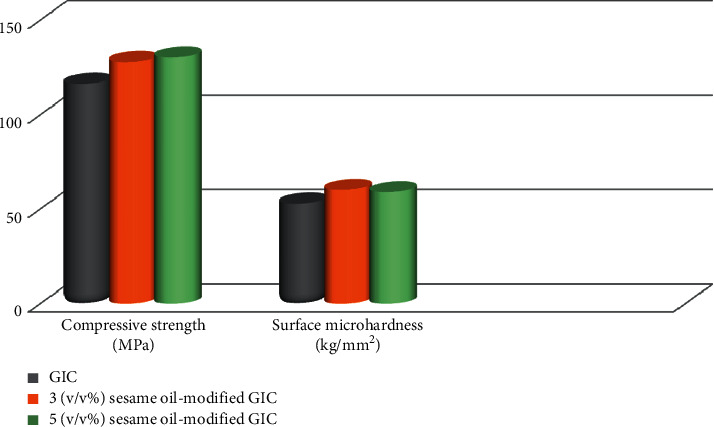
A graphical presentation of compressive strength, and surface micohardness of the studied groups.

**Figure 3:**
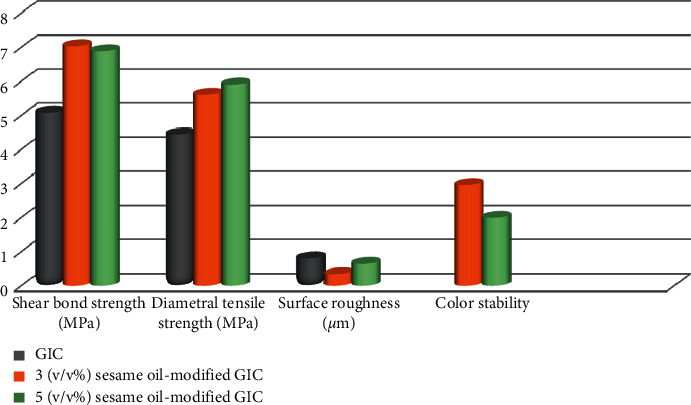
A graphical presentation of shear bond strength, diametral tensile strength, surface roughness, and color stability results of the studied groups.

Table 2 shows the color stability results. Student *t*-test revealed a significant difference (*p* ≤ 0.05) between the two tested groups, where 5 (v/v%) sesame oil-modified cement had higher mean (2.95 ± 0.3). The color difference mean values of both groups are clinically acceptable (Δ*E *≤ 3.3). A graphical presentation of compressive strength and surface microhardness is shown in Figure 2. Shear bond strength, diametral tensile strength, surface roughness, and color stability results are graphically presented in Figure 3.

## 4. Discussion

Right now, no dental material presented in the market has ideal characteristics for any dental application, so, the researchers are continuously searching for upgrading [14, 15]. According to this target, a combination of existing dental materials with a variety of other substances may be very promising. However, further research work has to be performed to suggest their appropriate application [16]. In an agreement with this attitude, sesame oil was selected for the current research due to its proven health benefits in dental field, for example, effectiveness in dental caries, dental plaque, and halitosis besides trouble-free availability [10, 11].

In FTIR spectra of GIC, fading of the bands at 1250 and 1710 cm^−1^ is an sign for aluminum polyacrylate formation [17]. The bands representing the acid base reaction are at 846 (Si-O-Al), 1085 and 627 (Si-O), and 457 (Si-O-Si) with the silica gel (Si-OH, OH stretching frequency) at 3452. Carboxylate groups (COO^−^) were recognized at 1589 and 1447. Al and Ca polysalts are characterized by bands at 1447 and 1409, respectively [18].

The peak around 1462 cm^−1^ proved that the reaction between the ingredients of the cement has been fulfilled into 24 hours [17]. The changes (in terms of difference in sharpness and shift of some peaks) within the chemical structure after modification of GIC by sesame oil are a gauge of the chemical interactions between sesame oil and GIC.

On the other hand, spectrum of sesame oil is similar to that of triglyceride. Absorbance band at 3007 cm^−1^ was caused by stretching vibration of (cis) C = CH, whilst the peak at 2953 cm^−1^ was due to asymmetric stretching vibration of methyl (−CH3) group. Both peaks at 2922 and 2853 cm^−1^ were originating from asymmetric and symmetric stretching vibrations of methylene (−CH2). At 1744 cm^−1^, the carbonyl (C = O) stretching vibration was observed, though the peak at 1654 is due to C = C stretching vibration. The bending vibrations of methylene and methyl were obvious at wave numbers of 1460 and 1376 cm^−1^, respectively. The absorbance bands at 1237, 1160, 1118, and 1098 cm^−1^ were from C-O vibrations. Moreover, peaks at 996 and 850 were a result of bending out of plane vibrations of –HC = CH– (trans) and –HC = CH– (cis), correspondingly [19, 20].

The null hypothesis was completely rejected, since sesame oil had the liability to alter the assessed physical properties of conventional GIC in both ratios positively: 3 and 5 (v/v%). Although the incorporation of some modifiers may adversely impact the physical properties of the parent material, here, the compressive and diametral tensile strengths of both 3 and 5 (v/v%) sesame oil-modified cement were significantly improved [21].

The potential explanation for that may be multifactorial. The composition of sesame oil is of a great importance while explaining the step-up of both compressive and diametral tensile strength. The oil contains considerable amount of minerals including calcium which may induce a higher degree of the acid base reaction. More Ca^2+^ ion may be available for polysalt bridge formation and cross-linking into Ca polyacrylate chains, in that way reinforcing the GIC matrix, and increasing the mechanical properties [22, 23]. Also, normally during gelation stage of the reaction, the carboxylate groups of the polyacrylic acid polymer chains become charged, repel each other, uncoil, and seemingly retain a more linear pattern. Sesame oil may augment the cross-linking of these polymer chains. Sesame oil may spread within the matrix formed increasing its cohesive strength in terms of more ionic cross-links, interlocking of chains and hydrogen bridges. What is more, sesame oil may serve as an adhesive occluding the pores within the matrix rendering more cohesive, stronger, and less brittle cement [21, 24–26]. The interactions seem to be more prominent in the concentration of 5 v/v% of sesame oil; however, the difference from the lower ratio (3 v/v%) of modification was insignificant.

Shear bond strength results may be controversial. The addition of sesame oil to the liquid may influence the density of free carboxylic groups necessary for the chemical bond to the tooth structure. The presence of Ca ions in sesame oil may exhaust some of the carboxylic acid in polysalt bridge formation. In opposition, another factor may elucidate the increase in the bond strength values. The phosphrous and calcium content of sesame oil may form chemical bond with calcium of the tooth structure rendering more adhesion [8, 9, 23, 27].

Rationally, the surface microhardness results should be consistent with the results of both compressive strength and diametral tensile strength. The ability of sesame oil to increase the degree of cross-linking and interlocking within the cement matrix may offer a stronger and harder cement with more resistance to scratching [24, 25]. Additionally, sesame oil is an excellent source of phosphorous, iron, magnesium calcium, manganese, copper, and zinc, and this mineral combination may be a complementary aspect while illuminating the oil potentiality to enhance the surface microhardness of the cement [22–25]. This reinforcing minerals' mixture can heighten the wear resistance as well as diminishing brittleness of the cement [28, 29]. The results are in harmony with the findings by Moshaverinia et al., who concluded that nanobioactive ceramics improved the mechanical properties of GIC and its bond strength to dentin [8]. Alternatively, the results are inconsistent with another study which revealed that the incorporation of bioactive glass in the glass ionomer cement deteriorated its mechanical properties [30].

It was claimed that viscous liquid of the conventional GIC may be a main cause of difficult manipulation and development of porosity during hand mixing. Additionally, it was reported that hand mixed cement has larger bubbles than encapsulated forms [31]. This porosity may be one of the causes for higher surface roughness value of the conventional cement. The findings of the surface roughness support the assumption that sesame oil or its ingredients may be responsible for sealing the pores within the cement in both tested concentrations with an enhancement of the packing density. However, the lower concentration of the sesame oil (3 v/v%) appears to be more appropriate for this task [32].

Sesame oil possesses distinctions in its color according to the seed from which it was extracted, white or black seeds. It may vary from pale yellow to golden, and in East Asia its color may extend to dark red [33]. In the existing study, the oil used to modify GIC was of a pale yellow color. Although both concentrations tested, 3 and 5 (v/v%), exhibited perceptible color changes (Δ*E* < 3.3), the result presented by the lower concentration was significantly different and more clinically satisfying [34].

## 5. Conclusions

Considering the findings of this study, the incorporation of sesame oil in glass ionomer cement generally improved its mechanical properties with an agreeable color change. Sesame oil may be an exceptional natural product with bioactive ingredients rendering the oil suitable for specific applications in dentistry. Although sesame oil may be a unique material for reinforcement of conventional GIC, nevertheless, further supporting studies to evaluate other properties, as fluoride release and water sorption and solubility, may provide a definite conclusion concerning this assignment.

## Figures and Tables

**Table 1:** Means, standard deviations and Tukey's analysis of the physical properties of the studied groups.

Group	Compressive strength (MPa)	Shear bond strength (MPa)	Diametral tensile strength (MPa)	Surface microhardness (kg/mm^2^)	Surface roughness (*μ*m)
Control (unmodified GIC)	116.42^b^ ± 3.02	5.08^b^ ± 0.68	4.44^b^ ± 0.32	52.84^b^ ± 1.22	0.8^a^ ± 0.09
3 (v/v%) sesame oil-modified GIC	128^a^ ± 1.92	7.03^a^ ± 0.38	5.6^a^ ± 0.17	60.08^a^ ± 0.65	0.34^c^ ± 0.07
5 (v/v%) sesame oil-modified GIC	130.34^a^ ± 4.15	6.84^a^ ± 1.02	5.9^a^ ± 0.53	58.9^a^ ± 0.91	0.64^b^ ± 0.04
*p*-value	≤0.0001	≤0.0001	≤0.0001	≤0.0001	≤0.0001

*∗*Means with the same superscript letter in each column are not significantly different at *p* ≤ 0.05. *∗*GIC: glass ionomer cement.

**Table 2:** Student *t*-test of the color stability results of the studied groups.

Groups	Color difference (Δ*E*)		
	Mean + SD	*t*-value	*p*-value
3 (v/v%) sesame oil-modified GIC	1.99 ± 0.24	5.63	0.0005
5 (v/v%) sesame oil-modified GIC	2.95 ± 0.3		

*∗* GIC: glass ionomer cement. Color difference (Δ*E*) ≤ 3.3 are clinically acceptable.

## Data Availability

All the data presented or analyzed during this study are included in this article.

## References

[1] Srinivasan K., Chitra S. (2015). Emerging trends in oral health profession: the biomimetic—a review. *Archives of Dental and Medical Research*.

[2] Lohbauer U. (2010). Dental glass ionomer cements as permanent filling materials?—Properties, limitations and future trends. *Materials*.

[3] Ab-Ghani Z., Ngo H., McIntyre J. (2007). Effect of remineralization/demineralization cycles on mineral profiles of fuji IX fast in vitro using electron probe microanalysis. *Australian Dental Journal*.

[4] Scholtanus J. D., Huysmans M.-C. D. N. J. M. (2007). Clinical failure of class-II restorations of a highly viscous glass-ionomer material over a 6-year period: a retrospective study. *Journal of Dentistry*.

[5] Lohbauer U., Walker J., Nikolaenko S. (2003). Reactive fibre reinforced glass ionomer cements. *Biomaterials*.

[6] Silva R. M., Pereira F. V., Mota F. A. P., Watanabe E., Soares S. M. C. S., Santos M. H. (2016). Dental glass ionomer cement reinforced by cellulose microfibers and cellulose nanocrystals. *Materials Science and Engineering: C*.

[7] Gurgan S., Kutuk Z. B., Ergin E., Oztas S. S., Cakir F. Y. (2017). Clinical performance of a glass ionomer restorative system: a 6-year evaluation. *Clinical Oral Investigations*.

[8] Moshaverinia A., Ansari S., Moshaverinia M., Roohpour N., Darr J. A., Rehman I. (2008). Effects of incorporation of hydroxyapatite and fluoroapatite nanobioceramics into conventional glass ionomer cements (GIC). *Acta Biomaterialia*.

[9] Moshaverinia A., Ansari S., Movasaghi Z., Billington R. W., Darr J. A., Rehman I. U. (2008). Modification of conventional glass-ionomer cements with N-vinylpyrrolidone containing polyacids, nano-hydroxy and fluoroapatite to improve mechanical properties. *Dental Materials*.

[10] Bedigian D., Harlan J. R. (1986). Evidence for cultivation of sesame in the ancient world. *Economic Botany*.

[11] Namiki M. (1995). The chemistry and physiological functions of sesame. *Food Reviews International*.

[12] Shanbhag V. K. L. (2017). Oil pulling for maintaining oral hygiene—a review. *Journal of Traditional and Complementary Medicine*.

[13] Al-Qahtani W. A., Sandeepa N. C., Abdullah E. K. (2020). A clinical study comparing the efficacy of SesameOil with desensitizing tooth paste in reducing dentinal hypersensitivity: a randomized controlled trial. *International Journal of Dentistry*.

[14] Jeevan S., Sindhu R., Manipal S., Prabu D., Mohan R., Bharathwaj V. V. (2019). Efficacy of oil pulling with sesame oil in comparison with other oils and chlorhexidine for oral health: a systematic review. *Journal of Pharmaceutical Sciences & Research*.

[15] McCabe J. F., Walls A. (2013). *Applied Dental Materials*.

[16] Powers J. M., Wataha J. C. (2014). *Dental Materials: Properties and Manipulation*.

[17] Young A. M., Sherpa A., Pearson G., Schottlander B., Waters D. N. (2000). Use of Raman spectroscopy in the characterisation of the acid-base reaction in glass-ionomer cements. *Biomaterials*.

[18] Yamakami S. A., Ubaldini A. L. M., Sato F., Medina Neto A., Pascotto R. C., Baesso M. L. (2018). Study of the chemical interaction between a high-viscosity glass ionomer cement and dentin. *Journal of Applied Oral Science*.

[19] Arslan F. N., Çağlar F. (2019). Attenuated total reflectance-fourier transform infrared (ATR-FTIR) spectroscopy combined with chemometrics for rapid determination of cold-pressed wheat germ oil adulteration. *Food Analytical Methods*.

[20] Ozulku G., Yildirim R. M., Toker O. S., Karasu S., Durak M. Z. (2017). Rapid detection of adulteration of cold pressed sesame oil adultered with hazelnut, canola, and sunflower oils using ATR-FTIR spectroscopy combined with chemometric. *Food Control*.

[21] Yesilyurt C., Er K., Tasdemir T., Buruk K., Celik D. (2009). Antibacterial activity and physical properties of glass-ionomer cements containing antibiotics. *Operative Dentistry*.

[22] Mittal S., Soni H., Sharma D., Mittal K., Pathania V., Sharma S. (2015). Comparative evaluation of the antibacterial and physical properties of conventional glass ionomer cement containing chlorhexidine and antibiotics. *Journal of International Society of Preventive and Community Dentistry*.

[23] Cheng F. C., Jinn T. R., Hou R. C., Tzen J. T. (2006). Neuroprotective effects of sesamin and sesamolin on gerbil brain in cerebral ischemia. *International Journal of Biomedical Science : IJBS*.

[24] Aoki H. (1991). *Science and Medical Applications of Hydroxyapatite*.

[25] Khoroushi M., Mansoori-Karvandi T., Hadi S. (2012). The effect of pre-warming and delayed irradiation on marginal integrity of a resin-modified glass-ionomer. *General Dentistry*.

[26] Pinto M. C. C., Gomes F. W., Melo C. K., Melo P. A., Castro M., Pinto J. C. (2012). Production of poly(acrylic acid) particles dispersed in organic media. *Macromolecular Symposia*.

[27] Lucas M. E., Arita K., Nishino M. (2003). Toughness, bonding and fluoride-release properties of hydroxyapatite-added glass ionomer cement. *Biomaterials*.

[28] Racoti A., Rusen E., Dinescu A (2016). Ginger essential oil encapsulation in PMMA microcapsules. part II. *UPB Scientific Bulletin Series B*.

[29] Kerby R. E., Bleiholder R. F. (1991). Physical properties of stainless-steel and silver-reinforced glass-ionomer cements. *Journal of Dental Research*.

[30] De Caluwé T., Vercruysse C. W. J., Ladik I. (2017). Addition of bioactive glass to glass ionomer cements: effect on the physico-chemical properties and biocompatibility. *Dental Materials*.

[31] Williams J. A., Billington R. W. (1991). Changes in compressive strength of glass ionomer restorative materials with respect to time periods of 24 h to 4 months. *Journal of Oral Rehabilitation*.

[32] Mitchell C. A., Douglas W. H. (1997). Comparison of the porosity of hand-mixed and capsulated glass-ionomer luting cements. *Biomaterials*.

[33] Morris J. B., Janick J., Whipkey A. (2002). Food, industrial, nutraceutical, and pharmaceutical uses of sesame genetic resources. *Trends in New Crops and New Uses*.

[34] Yap A. U., Sim C. P., Loh W. L., Teo J. H. (1999). Human-eye versus computerized color matching. *Operative Dentistry*.

